# Impact of Treatment Methods on the Surface Properties of the Mg-Containing Zeolite Y

**DOI:** 10.3390/ma18051033

**Published:** 2025-02-26

**Authors:** Andrzej Biessikirski, Grzegorz Piotr Kaczmarczyk, Malwina Kolano, Karolina Kaznowska-Opala, Małgorzata Ruggiero-Mikołajczyk, Jacek Gurgul, Łukasz Kuterasiński

**Affiliations:** 1 Faculty of Civil Engineering and Resource Management, AGH University of Krakow, 30-059 Krakow, Poland; grzegorz.kaczmarczyk@agh.edu.pl (G.P.K.); mkolano@agh.edu.pl (M.K.); kazn@agh.edu.pl (K.K.-O.); 2 Jerzy Haber Institute of Catalysis and Surface Chemistry, Polish Academy of Sciences, 30-239 Krakow, Poland; malgorzata.ruggiero-mikolajczyk@ikifp.edu.pl (M.R.-M.); jacek.gurgul@ikifp.edu.pl (J.G.)

**Keywords:** zeolite, surface, roughness, magnesium, porosity

## Abstract

In the undertaken research, we investigated the preparation route’s influence mainly on the surface properties of the final form of Mg-containing zeolite Y. The parent zeolite was subjected to modification with aqueous solutions of magnesium nitrate via impregnation, ion-exchange, and ultrasonic techniques, respectively. The results obtained from the Atomic Force Microscopy (AFM), Computer Tomography (CT), and crystallinity evaluations indicated that the method of zeolite modification influenced the physicochemical properties of the studied samples. Wet impregnation caused additional surface roughness, whereas both ion-exchange and sonication led to surface smoothing of the Mg-containing zeolite Y. Nitrogen adsorption analysis indicated no enormous changes in the porosity of Mg-containing zeolite Y, which can be explained by a relatively high resistance of zeolite to interaction with magnesium nitrate aqueous solutions. However, the biggest changes in porosity were observed for Mg-Y prepared via the impregnation technique due to the longest contact between the zeolite and Mg solution.

## 1. Introduction

Zeolites are microporous crystalline aluminosilicates, whose framework is created by a corner-sharing TO_4_ (T = Si and Al, among others) tetrahedral arranged in a 3D structure [1]. This group of materials has wide industrial applications, e.g., catalysts in acid-catalyzed reactions in petrochemistry, oil refining, coal chemicals, producing fine chemicals, and environmental protection [2,3]. The modification of physicochemical properties of prepared zeolites can be realized by the post-synthesis modification of microporous systems, i.e., by impregnation [4,5,6], ion-exchange [7,8,9], or sonication [10,11,12,13,14].

Impregnation of zeolites followed by their thermal treatment allows one to introduce metal sites as oxides (M_x_O_y_) or cations (M^n+^) without apparent deterioration of a zeolite-based sample crystalline structure [4,5]. Simultaneously, the production of metal sites as metallic-phase (M^0^) is impossible due to the presence of oxygen under treatment conditions, as well as the absence of reagents reducing oxidized metal sites. However, the chemical form of oxidized metal depends on its content in zeolite and the form of zeolite carrier: protonic (H-zeolite) or sodium (Na-zeolite). The metal–zeolite systems obtained from H-zeolite mainly contain cations, whereas the counterparts derived from the Na-zeolite reveal much higher contents of oxides, and this was reported in one of our previous works [6].

Ion-exchange belongs to the oldest method of incorporating the metal sites inside the porous structure of zeolite. Typically, the ion-exchange procedure is conducted in an aqueous solution via several treatment cycles with an excess of the desired cation followed by washing. In this method, zeolites and metal precursors (most often in the form of aqueous solutions of their salts) are blended at high temperatures to ensure efficient diffusion of cations inside the pores leading to their uniform distribution in the whole volume of zeolite carrier compared to the impregnation technique [7,8]. Metal-containing zeolites prepared via the ion-exchange method were often used as active and selective catalysts for DeNO_x_ processes [9].

Sonication is a fairly new method based on ultrasonic cavitation in a liquid environment leading to unique reaction pathways (inaccessible for other preparation methods), which allows them being used to develop new methods of material synthesis and/or post-synthesis modification [10]. In the context of post-synthesis modification of zeolites, sonication resulted in more significant effects than conventional techniques. For instance, Hosseini et al. [11] conducted ultrasonic-assisted dealumination of zeolite Y. In turn, Zhang et al. [12], Oruji et al. [13], and Khoshbin and Karimzadeh [14] modified microporous zeolites in the presence of ultrasound to obtain hierarchical zeolite systems. These materials revealed a higher dealumination or more intensive formation of mesoporosity than the samples modified under conventional conditions. Furthermore, these materials were more efficient for catalytic purposes.

In our previous research [15], we investigated Mg-containing zeolite Y of Si/Al of 2.65 as a modifier of ANFO (Ammonium Nitrate Fuel Oil)-based energetic materials. We indicated that the addition of Mg-Y caused improved energetic properties of such modified ANFO-based materials with a generally reduced volume of post-decomposition fumes (CO_x_ + NO_x_). However, ANFO’s velocity of decomposition (VOD) depended on the method of zeolite Y modification. Namely, Mg-Y prepared via the impregnation method resulted in the growth of VOD, whereas ultrasonically prepared Mg-Y caused the opposite effect. Interestingly, Mg-Y prepared using the ion-exchange technique had an ambiguous effect on the energetic properties of the final form of ANFO-based energetic materials. The published findings focused mainly on the physicochemical properties of ANFO-type materials, including their energetic performance.

Compared to our previous studies [15], the center of gravity in the current considerations is shifted towards a more profound insight into the physicochemical properties of variously prepared Mg-Y zeolite. This connection between the current studies and previous research [15] constitutes some kind of “bridge” allowing us to add to existing knowledge about the physicochemical properties of all ingredients creating ANFO and the energetic performance of such a type of material.

## 2. Materials and Methods

### 2.1. Materials

A Na-Y-type zeolite (Si/Al = 2.65) acting as a reference sample was provided by Mątwy (Inowrocław, Poland). The sample was modified with magnesium via impregnation, ion-exchange, and ultrasonic-assisted impregnation techniques.

Magnesium-impregnated zeolite (Mg-Y-impr) was prepared by wet impregnation method using 6 g of dry Na-Y zeolite and 5.33 g of Mg(NO_3_)_2_·6H_2_O (Sigma-Aldrich, St. Louis, MO, USA, ACS grade > 99%) diluted in 6 g of distilled water, followed by drying at 80 °C for 12 h.

Magnesium Ion-Exchanged Zeolite (Mg-Y-ion-exch) was prepared through five cycles of Na⁺/NH₄⁺ ion-exchange on sample 1, using 500 mL of 0.5 M aqueous magnesium nitrate solution at 80 °C for 2 h with 30 g of the parent zeolite.

Ultrasonic-assisted impregnation (Mg-Y-son) was prepared with 5 g of sample 1 immersion in 200 mL of 0.5 M aqueous magnesium nitrate solution. The sample was subjected to ultrasound (QSonica Q-700, Newtown, CT, USA, ½” diameter horn, 60 W, 20 kHz) for 30 min. The mixture was maintained at room temperature during sonication in an ice bath.

All zeolite samples were subjected to a final treatment of triple centrifugation at 4200 rpm for 10 min, drying at 80 °C for 12 h, and calcination at 500 °C for 4 h under an airflow of 50 mL/min.

### 2.2. Methods

Zeolite surface analysis was conducted with an NT-MDT Solver BIO atomic force microscope (AFM) (NT-MDT, Moscow, Russia) fitted with an SMENA SFC050L scanning head (Van Nuys, CA, USA). Scanning was carried out in air under semicontact mode. Image graphical processing was completed using the proprietary software provided by the microscope manufacturer.

The root mean square (RMS) topographical parameter determined the roughness of the zeolite particle layers, was performed using the ex situ AFM method, and was calculated using Gwyddion 2.56 software. A detailed description of the RMS technique was reported in [16].

Zeolite crystallinity was determined based on XRD experiments using a PANalytical X’Pert PRO MPD diffractometer (Malvern, UK, 40 kV and 30 mA), equipped with a CuKα generator (λ = 1.5418 Å). The 2θ angle was in the range of 5–50°, with a 0.033° step. The zeolite samples were in the form of powder and were placed in holders. The calculations of the average size of crystallites were based on the Scherrer equation and were conducted via PANanalytical X Pert Data Viewer software (https://ikifp.edu.pl/en/structure/laboratories/xrd-and-thermoanalysis-laboratory/?acf-label=aparatura) connected with a diffractometer.

Computed tomography (CT) imaging was conducted using a GE Phoenix M scanner (General Electric Company, Hürth, Germany) equipped with two distinct types of X-ray tubes: microfocus and nanofocus. Given the small dimensions of the sample, the nanofocus tube was selected for this study due to its capability to image minute objects with high precision. The nanofocus tube is specifically engineered for stable, low-power X-ray generation, enabling high magnification and detailed resolution down to 1 µm. Scans were conducted at a voltage of 50 kV and a current of 40 µA, with the nanofocus tube mode set to “1”. The sample, a fine powder, was placed inside a specially drilled cavity within a low-absorbance foam material to minimize background interference. A magnification factor of 100× was achieved and the resulting voxel size was 2^3^ µm^3^. Each scan targeted a single clump of material, capturing 2700 X-ray projections. Data reconstruction was executed using GE’s dedicated software, datos|x 2, incorporating Automatic Geometry Calibration (AGC) and Beam Hardening Correction (BHC+), with a correction intensity of 6.7. The reconstructed tomographic images were rendered in 14-bit grayscale, where variations in grayscale intensity correlated with material density—brighter regions indicated areas of higher X-ray absorbance, signifying increased density.

Low-temperature nitrogen adsorption was conducted using an Autosorb-1 instrument (Boynton Beach, FL, USA) at −196 °C. The S_Langmuir_ was calculated based on the Langmuir equation. Furthermore, the t-plot, Dubinin–Radushkevich (DR), and Horvath–Kavazoe (HK) methods were used to determine the pore volume and size distribution. The chosen techniques for the porosity characterization of the prepared samples corresponded to their microporous character [17]. Before measurement, each sample was degassed at 250 °C for 20 h.

Diffuse reflectance UV–vis analysis (DR-UV-ViS) was conducted using a high-resolution AvaSpec-ULS3648 spectrometer (Avantes, Apeldoorn, The Netherlands) equipped with a high-temperature reflection probe (FCR-7UV400–2-ME-HTX, MT Brandão, Porto, Portugal, 700 μm fibers) and a Praying Mantis High-Temperature Reaction Chamber (Harrick Scientific Co., Ossining, NY, USA). The AvaLight-D(H)-S Deuterium Halogen light was used as a light source. The DR-UV-ViS spectra were recorded at 200–700 nm. The instrument was equipped with AvaSoft v 9.0 software. Directly before analysis, all samples were dehydrated at 50 °C in air for 1 h.

The X-ray photoelectron spectroscopy (XPS) was used for the determination of the chemical form of magnesium existing in the prepared sample. The Al Kα (1486.6 eV) X-ray source with the anode operating at 12 kV and 10 mA current emission was applied to generate core excitation. The hemispherical analyzer (SES R4000, Gammadata Scienta, Uppsala, Sweden) was applied. The energy resolution of the spectrometer was 0.9 eV for the pass energy of 100 eV.

The status of Al in the prepared materials was determined by the solid-state ^27^Al MAS NMR (Magic Angle Spinning Nuclear Magnetic Resonance) technique using a Bruker Advance III 500 MHz WB spectrometer (Billerica, MA, USA, magnetic field of 11.7 T and basic resonance frequency of 130.3 MHz, at a spinning rate of 12 kHz) in zirconia rotors, using high-power proton decoupling (SPINAL64), with 0.2 μs (π/12) pulses and a recycle delay of 0.1s. The chemical shifts of ^27^Al MAS NMR were referenced to 1M aqueous Al(NO_3_)_3_.

Analogously, the silicon status in the studied samples was investigated by the solid-state ^29^Si MAS NMR technique using the same equipment, but under different conditions. Namely, the basic resonance frequency and spinning rate in zirconia rotors were 99.4 MHz and 8 kHz, respectively. High-power proton decoupling (SPINAL64), with 5.8 μs (π/3) pulses and a repetition time of 20 s, was used. The chemical shifts of ^29^Si MAS NMR were linked to Tetramethylsilane (TMS; >99%). The Si/Al ratio for all studied samples was determined by formula [18] based on the appearance of ^29^Si MAS NMR bands.

The thermal stability of the prepared zeolite samples was investigated by Thermogravimetry (TG) and Differential Scanning Calorimetry (DSC) using NETZSCH STA 409 PC/PG (Selb, Germany) at 20–1000 °C with a temperature ramp of 10 °C∙min^−1^ under an airflow of 30 mL∙min^−1^. The sample weight was 20 mg whereas the TG drift was ca. 5 μg, corresponding to 0.02 mass%.

## 3. Results and Discussion

**Figure 1:**
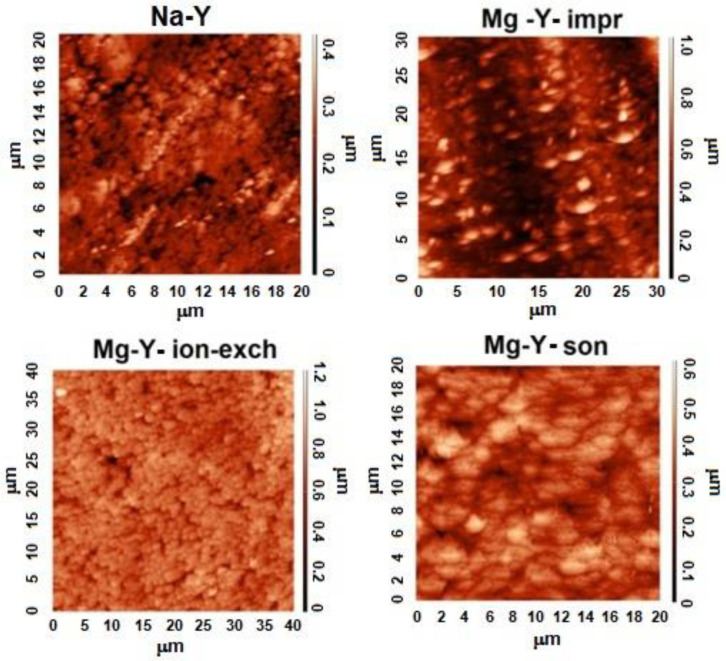
AFM images of variously modified zeolite Y. Adapted from [15]. Copyright @2022. Based on the CC-BY-4.0 license.

The AFM research method, Figure 1, can be used to evaluate zeolite surfaces under molecular resolution [19,20].

Figure 1 illustrates that the surface of reference Na-Y zeolite was uniform, with octahedral grains displaying a length of 1–3 μm and a height of up to 1 μm. Modifications to the parent zeolite surface demonstrated the impact of different synthesis methods. The zeolite surfaces remained uniform for both the ion-exchange and sonication methods. In contrast, sample Mg-Y-impr indicated a highly differentiated surface [15].

The measured roughness of the surface of all prepared zeolite samples was expressed by the root mean square (RMS) topographical parameter from the AFM technique [16]. The RMS results are summarized in Table 1.

Upon first glance, it may be concluded that the way Mg-Y zeolite is prepared has a relevant impact on the roughness of the final form of the zeolite sample. Mg-Y prepared via wet impregnation was characterized by a higher RMS value (0.235 µm) than the reference sample Na-Y (0.189 µm). The introduction of magnesium into zeolite Y by either ion-exchange (Mg-Y-ion-exch) or ultrasonic irradiation (Mg-Y-son) caused a reduction in the RMS values (0.162 µm or 0.156 µm, respectively).

No characterization of the roughness of similar systems (Mg/zeolite) was found in the bibliography. In the case of our Mg-Y zeolite samples, the amount of Mg mainly influenced their roughness (Table A1), whereas the magnesium chemical form played a supporting role in the status of the surface of the synthesized Mg-Y samples. However, it seems that MgO has a dominating role in the creation of the roughness of the Mg-Y surface.

**Figure A1:**
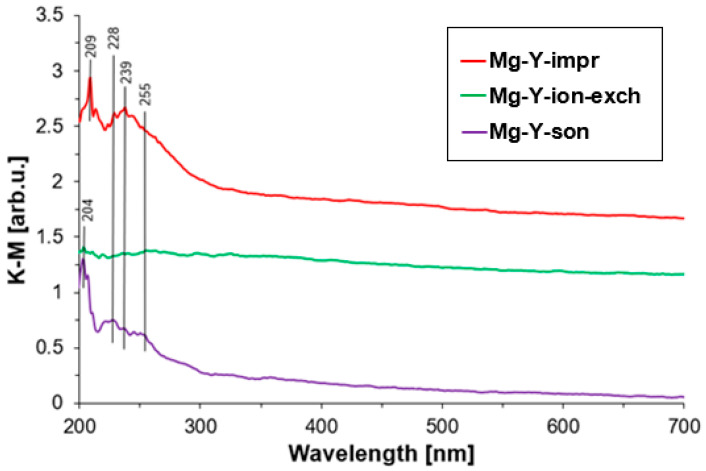
DR UV-ViS spectra of the variously modified Y-type zeolites.

**Figure A2:**
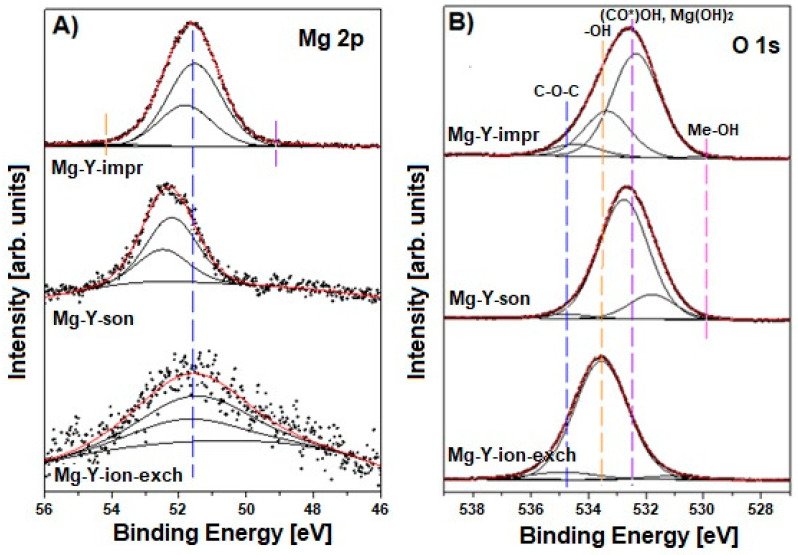
XPS spectra of the variously modified Y-type zeolites: (**A**) Mg 2p3/2; (**B**) O 1s.

The chemical form of magnesium in various Mg-Y samples was studied in our previous research by the FT-IR technique [15]. In our current studies, we confirmed our findings on the Mg status via DR UV-Vis and XPS analyses (Figure A1 and Figure A2).

In the case of the impregnation method, Mg was present as MgO, as evidenced by a distinct IR band at 1400 cm^−1^ in [15] or based on the DR UV-ViS spectra (the band at 225–260 nm [21])—Figure A1. In our parent zeolite in the sodium form, during impregnation, the following reaction took place: Na-Y + magnesium nitrate → Mg-Y + sodium nitrate. In this case, NaNO_3_ was not removed during calcination, which shifted the reaction equilibrium towards Na-Y. This caused limited consumption of magnesium nitrate during the zeolite modification, which in combination with high-temperature treatment (calcination) led to the formation of magnesium oxide. In the case of zeolite additives containing Mg introduced via ion-exchange, the bands assigned to MgO were not found. In the case of Mg-Y zeolite prepared via the ion-exchange method, Mg is present as a divalent cation caused by the reaction occurring in this procedure, as follows: Na-Y + magnesium nitrate → Mg-Y + sodium nitrate, where NaNO_3_ is fully removed during the centrifugation followed by the calcination.

In turn, for Mg-Y zeolite prepared using sonication, much weaker bands of MgO in relation to Mg-Y zeolite were found via the impregnation technique, which indicated the co-existence of magnesium in both cationic and oxide forms for sonochemically prepared Mg-Y.

In turn, the XPS results (Mg 2p and O 1s spectra) depicted in Figure A2 delivered new information on the interaction between Mg sites embedded in the zeolite Y carrier and the external environment. First of all, the occurrence of reduced metallic Mg^0^ species was excluded based on the absence of the bands at 49.5 eV [22]. This observation can be explained easily by the permanent contact of magnesium with oxidizing surroundings preventing the generation of Mg in metallic form. Interestingly, the absence of the bands attributed to the MgO phase was also observed (at 50.8 eV for Mg 2p and 530.4 eV for O 1s spectra, respectively), which upon first glance could be against UV-ViS data, particularly for the Mg-Y-impr sample (Figure A1). Nevertheless, further analysis of XPS spectra indicated a simultaneous presence of the signals in the binding energy range of 51.6–52.5 eV (from Mg 2p spectra) or 531.5–533.8 eV (from O 1s spectra), which corresponded to Mg-OH bonds from Mg(OH)_2_ groups as well as MgCO_3_ [22,23,24]. The existence of magnesium in the form of hydroxide or carbonate is correlated with its cationic form (Mg^2+^). However, finding the answer to a crucial question on the provenance of the XPS bands attributed to carbonates seems to be urgent. It must be underlined that we did not use carbon-containing ingredients during our sample preparation procedure. Fortunately, the research reported by Zimowska et al. [25] helped us to explain the appearance of the signals related to bonds, in which carbon was present. It turned out that magnesium incorporated into the aluminosilicate structure was responsible for the adsorption of CO_2_ from the air, which led to the formation of carbonates. Therefore, we did not observe the bands originating from MgO. Hence, it may be concluded that XPS results did not contradict observations taken from UV-Vis spectra but rather enriched our knowledge of the chemical form of magnesium with the awareness of the interaction between CO_2_ and Mg sites present in aluminosilicates (also zeolites).

**Figure 2:**
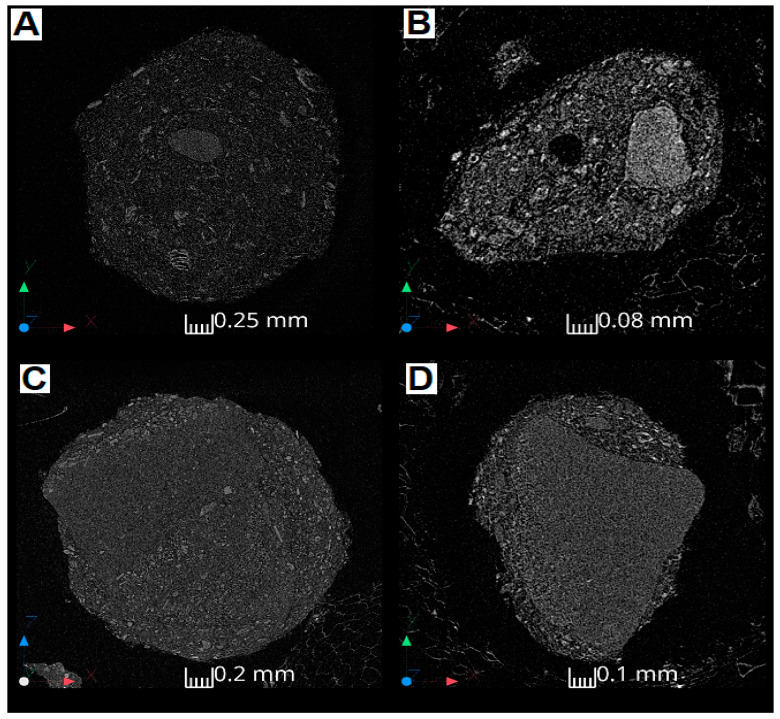
Tomography scans of zeolite: (**A**) Na-Y; (**B**) Mg-Y-impr; (**C**) Mg-Y-ion-exch; (**D**) Mg-Y-son.

**Figure A3:**
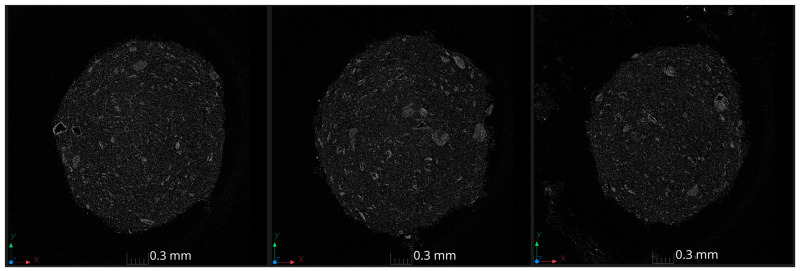
Tomography scans of zeolite Na-Y.

**Figure A4:**
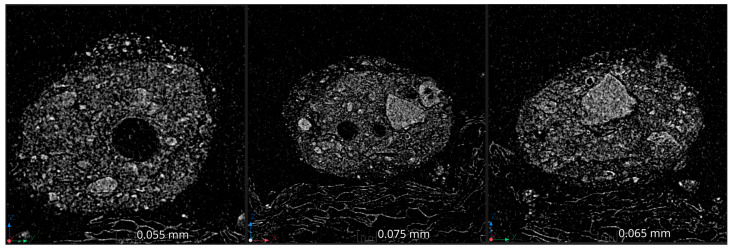
Tomography scans of zeolite: Mg-Y-impr.

**Figure A5:**
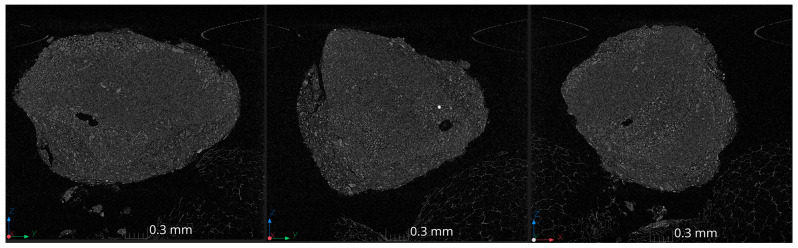
Tomography scans of zeolite: Mg-Y-ion-exch.

**Figure A6:**
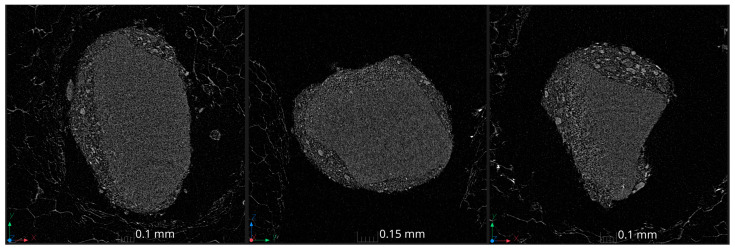
Tomography scans of zeolite: Mg-Y-son.

Computer Tomography images allowed us to illustrate the appearance of the grains of variously modified Y-type zeolite samples. The CT images are given in Figure 2, Figure A3, Figure A4, Figure A5 and Figure A6.

The Na-Y parent zeolite exhibited the largest dimensions among the analyzed samples, with a maximum diameter of grain exceeding 2.3 mm, Figure 2A. The grain surface was heterogeneous with existing high-density regions varying in size up to 0.4 mm. Notably, some of these high-density regions contained localized small hollow areas. In the Mg-Y impr sample, the material demonstrated significantly smaller grain dimensions, not exceeding 0.65 mm. The internal structure was more heterogeneous than the parent Na-Y zeolite and was characterized by numerous fractures; see Figure 2B. A prominent feature included a single central pore approximately 150 µm in diameter, contributing to structural weakening. The occurrence of this pore (resembling hole) may be caused by more aggressive conditions of Na-Y treatment with concentrated aqueous magnesium nitrate of acidic pH. It can lead to more intensive dealumination [26,27] followed by possible removal of adjacent singular Si atoms [28]. The sample Mg-Y-impr also displayed notable density variations and heterogeneity, with a distinct inclusion observed on the right side of Figure 2B. This high heterogeneity of the Mg-Y-impr sample corresponds with its elevated roughness. For a representative sample of Mg-Y-ion-exch, illustrated in Figure 2C, the maximal dimension was approximately 1.7 mm. The appearance of this sample grain suggests a much more uniform surface than in the case of two previous samples leading to lower roughness. Ultrasonic-assisted impregnation zeolite (Mg-Y-son) reveals two distinct structural fractions. A homogeneous central region is partially coated by an outer layer up to 250 µm thick, which displays an irregular structure. Fractures are absent within the homogeneous core but appear locally near the surface, which itself is uneven. The transition from the homogeneous to the heterogeneous structure may be either abrupt, as observed at the top of Figure 2D, or gradual, as seen on the left side of Figure 2D. The maximum dimension recorded for this group was 1.4 mm. The majority of the grain surface of the Mg-Y-son sample resembles sample Mg-Y-ion-exch. This feature is in line with their similar roughness. The obtained CT results correspond strongly with crystallinity calculations (Table 1). The highest change in average crystallite size as a result of the introduction of magnesium into Na-Y zeolite was found for the Mg-Y-impr sample. For this sample, the interaction between parent zeolite and impregnating magnesium nitrate solution led to a decrease in crystallite size from 897 Å (Na-Y) to 677 Å (Mg-Y-impr). Interestingly, the ion-exchange procedure (Mg-Y-ion-exch) practically did not alter crystal size (892 Å), whereas ultrasonic-assisted treatment (Mg-Y-son) only slightly reduced crystallite size (833 Å). Analogical trends were observed for other grains, depicted in Figure A3, Figure A4, Figure A5 and Figure A6.

**Figure A7:**
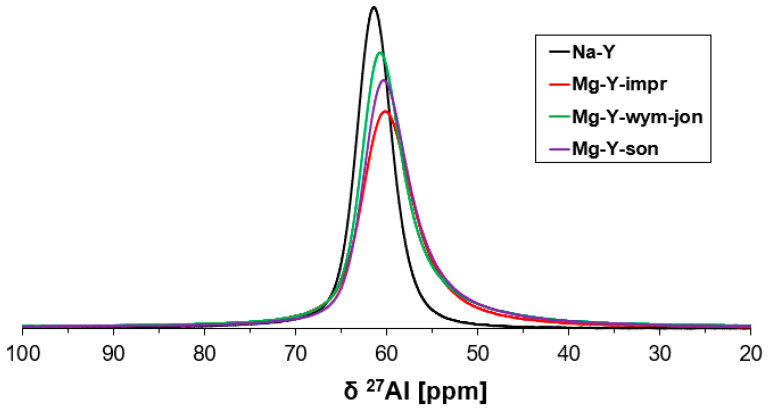
The ^27^Al MAS NMR spectra illustrating the influence of the Mg-Y preparation route on the chemical form and amount of Al.

**Figure A8:**
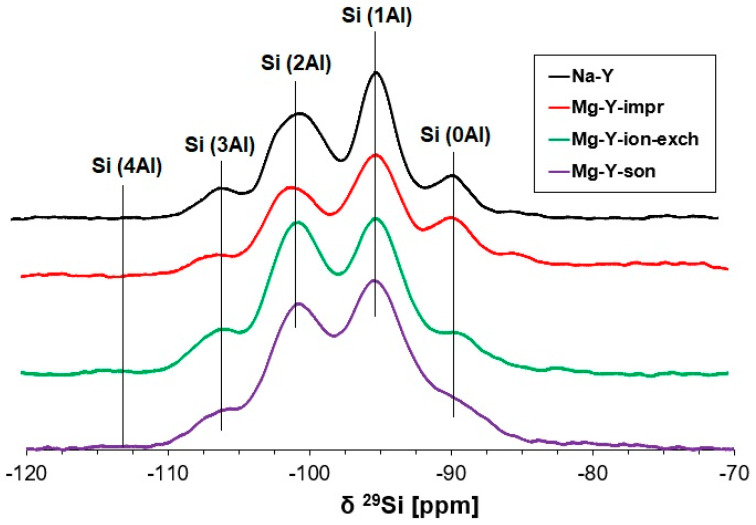
The ^29^Si MAS NMR spectra reflecting the influence of the Mg-Y preparation route on the chemical surrounding of Si.

The aforementioned dealumination of zeolite Y caused by the treatment of parent Na-Y sample with aqueous magnesium nitrate solutions was evidenced by the ^27^Al MAS NMR and ^29^Si MAS NMR spectra depicted in Figure A7 and Figure A8, respectively. From the comparison of signal intensities assigned to aluminum between studied samples, the slight scale of dealumination compared to the parent Na-Y sample can be seen (Figure A7). Nevertheless, the most apparent effect was found for zeolite modified with Mg via wet impregnation (Mg-Y-impr), whereas ion-exchange caused the lowest changes in the appearance of ^27^Al MAS NMR spectra. A middle effect was found for the sample prepared via the sonication technique (Mg-Y-son). Another observed result of the interaction between zeolite Y and aqueous magnesium nitrate solution was the lowering of chemical shift ^27^Al [ppm] values. This can be related to the partial migration of aluminum from lattice to extra-lattice positions [29].

Based on the appearance of the ^29^Si MAS NMR spectra (Figure A8), we calculated Si/Al ratios for the studied samples using the formula given in [18]. For comparison, we calculated Si/Al ratios from the EDS analysis reported in our previous paper [15]. The Si/Al ratios are summarized in Table A1. The Si/Al values obtained for the Mg-Y samples were predominantly lower than for the parent Na-Y sample independently of the applied technique (^29^Si MAS NMR vs. EDS). This observation did not indicate direct dealumination. However, for Mg-Y-impr, the Si/Al rose from 2.72 to 2.80 (by EDS); meanwhile, in the case of Mg-Y-son, the Si/Al increased from 2.40 to 2.49 (by ^29^Si MAS NMR).

Our results on the dealumination of zeolite Y by the exposure of parent Na-Y to acidic aqueous magnesium nitrate solution indicated an unambiguously milder character in comparison with the research in which zeolites were treated directly with mineral acid solutions. The effect of the change in Si/Al ratio depending on the zeolite topology and treatment conditions with mineral acids was reported in [30,31] and is summarized in Table 2. In our case, such weak effects in the increase in Si/Al ratio were within the measurement error implied from relatively high pH values (somewhat less than 4.0) of the used hydrated magnesium nitrate solutions compared to applied agents [30,31], where the pH reached values lower than 1.

**Figure 3:**
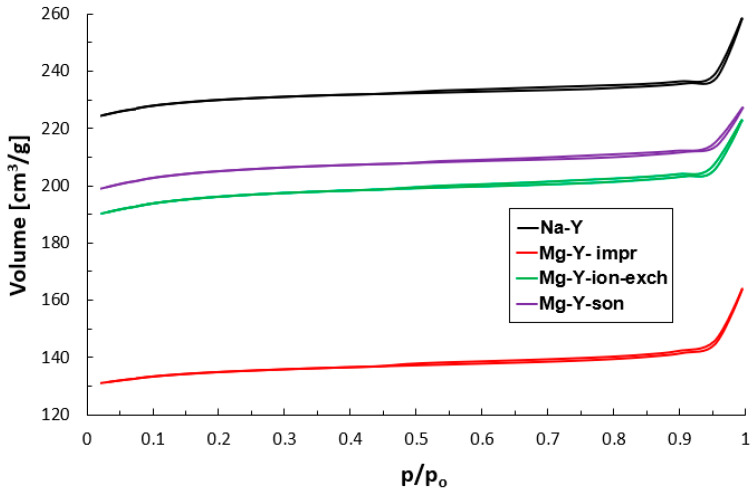
Adsorption–desorption N_2_ isotherms at −196 °C for the studied samples.

Table 3 and Figure 3 summarize the prepared samples’ porosity data. The appearance of nitrogen adsorption–desorption isotherms allowed us to classify them as a mixed type between Langmuir isotherms (I-type) typical of microporous materials and IV-type isotherms due to the occurrence of a thin hysteresis loop belonging to H4, suggesting the mesoporous character of the prepared samples [32]. It must be pointed out that the presence of hysteresis loops originates from the intercrystalline pores between the zeolite crystals, which was confirmed by tomography images (Figure 2). This phenomenon was reported for other microporous zeolite materials by Jong et al. [33], Hasan et al. [34], and Verboekend et al. [35].

Analysis of the quantitative data describing the porous structure of the synthesized zeolite samples (Table 3) led to the conclusion that all the studied materials were typically microporous, which prompted us to interpret the isotherms rather as a Langmuir type; however, small differences between micropore and total pore volume suggested very small mesopore content. The parent Na-Y zeolite was characterized by the Langmuir surface area (S_Langmuir_), total pore volume (V_total_), micropore volume (V_micro_), and an average pore size diameter (D) of 1012 cm^2^/g, 0.401 cm^3^/g, 0.364 cm^3^/g, and 11.5 Å, respectively.

The modification of parent zeolite Y with aqueous magnesium nitrate solution influenced the porosity of the studied Mg-Y samples slightly. The impregnation of the Na-Y zeolite with magnesium (Mg-Y-impr) resulted in a significant drop in Langmuir surface area from 1012 m^2^/g to 580 m^2^/g and a decrease in total pore volume from 0.401 cm^3^/g to 0.252 cm^3^/g (the micropore volume decreased analogously from 0.364 cm^3^/g to 0.212 cm^3^/g) with simultaneous growth of average pore diameter from 11.5 Å to 15.2 Å. These changes may originate from weak dealumination caused by the exposure of zeolite Na-Y to acidic impregnating aqueous Mg(NO_3_)_2_*6H_2_O solution. Magnesium nitrate in aquatic medium undergoes hydrolysis according to the following reaction: Mg^2+^ + 2H_2_O = Mg(OH)_2_ + 2H^+^. Hence, this type of solution is characterized by acidic properties. These changes in porosity observed for the Mg-Y-impr sample correspond with roughness, crystallinity, and Computer Tomography measurements.

The treatment of parent zeolite Na-Y with aqueous magnesium nitrate solution via ion-exchange procedure (Mg-Y-ion-exch) caused a small reduction in Langmuir surface area (from 1012 m^2^/g to 866 m^2^/g), total pore volume (from 0.401 cm^3^/g to 0.346 cm^3^/g), and micropore volume (from 0.364 cm^3^/g to 0.313 cm^3^/g). This method of introducing Mg into parent zeolite Y resulted in small growth in the average pore diameter (from 11.5 Å to 13.4 Å).

The application of ultrasonic irradiation during the modification of parent zeolite with magnesium nitrate solution (Mg-Y-son) also slightly affected the porosity of such prepared material. For this sample, Langmuir surface area, total pore volume, and micropore volume decreased from 1012 m^2^/g to 905 m^2^/g, from 0.401 cm^3^/g to 0.352 cm^3^/g, and from 0.364 cm^3^/g to 0.326 cm^3^/g, respectively. Milder changes in the porosity of the samples prepared in the presence of ultrasounds compared to analog and prepared via ion-exchange procedure are mainly due to the greater impact of ultrasounds on magnesium than on the zeolite carrier, which was also in contact with them [36], and may also be due to a better distribution of the magnesium sites on the zeolite carrier.

Our current results are in line with the research published previously in [37], where we investigated the porous structure of variously prepared Cu-containing zeolites of ZSM-5 and Y structure. In the cited study [37], we used aqueous Cu(NO_3_)_2_*3H_2_O solutions of similar pH (3.4) to our present research (3.8), which guaranteed reliable comparison. Furthermore, copper nitrate in aquatic medium undergoes hydrolysis according to the following reaction: Cu^2+^ + 2H_2_O = Cu(OH)_2_ + 2H^+^. Hence, Cu aqueous solution is also characterized by acidic properties (similar to Mg). The results of porosity studies for Cu-ZSM-5 zeolite indicated differences in the average pore size, which were lower for sonicated samples, and these were correlated with a better distribution of copper in the case of sonochemically prepared materials. In the case of NaY zeolite, the treatment with an aqueous copper nitrate solution led to significant changes in the porous structure in the systems based on this zeolite, with more pronounced effects observed for sonicated samples. The introduction of copper with the use of ultrasound led to the formation of a sample of a lower specific surface area compared to the counterpart modified by the ion-exchange procedure.

Generally, no enormous changes in the porosity of Mg-containing zeolite Y can be explained by a relatively high resistance of zeolite to interaction with magnesium nitrate aqueous solution of acidic character. The highest changes in porosity were observed for sample Mg-Y-impr, which was related to the longest contact between zeolite and Mg solution needed for the entire evaporation of this solution from the zeolite. Another factor was the increasing concentration of magnesium nitrate as a result of water evaporation leading to a decrease in pH, which was impossible to measure. It is commonly known that contact with an acid medium leads to the Al release from zeolite, particularly for the zeolites with a low Si/Al ratio [38,39,40,41].

**Figure A9:**
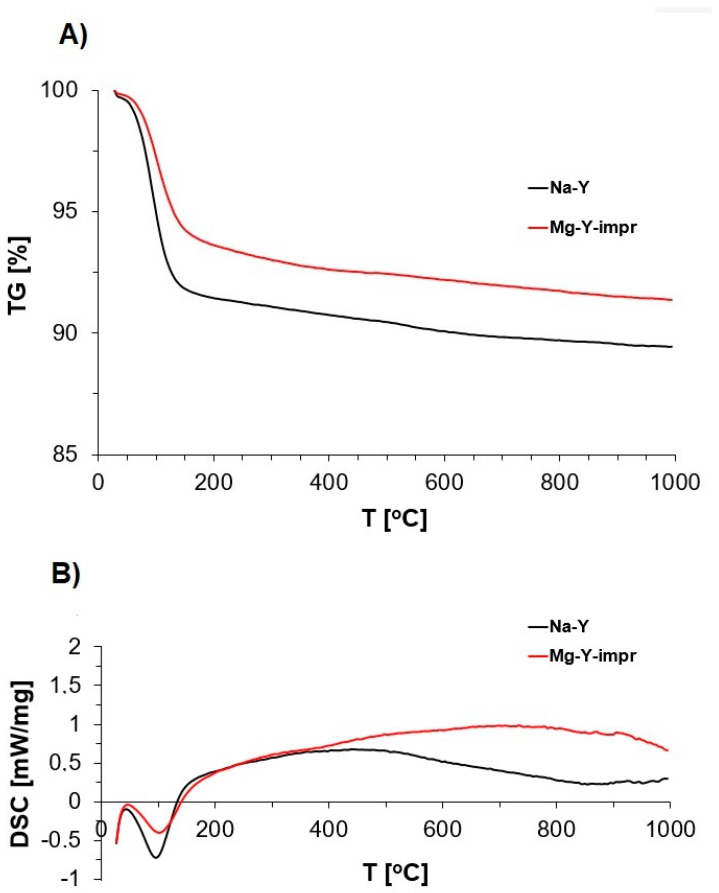
The influence of the presence of Mg in zeolite Y on its thermal stability: (**A**) TG curves; (**B**) DSC profiles.

For two chosen samples (Na-Y and Mg-Y impr), we determined thermal stability using Thermogravimetry (TG) and Differential Scanning Calorimetry (DSC). The results from thermal analysis are given in Figure A9. The appearance of both TG curves and DSC profiles evidenced that the treatment of parent zeolite (Na-Y) with Mg aqueous solutions did not deteriorate the thermal stability of the studied samples in the whole temperature range. The only effect was water evaporation from zeolites, which originated from the hydrophilic properties of this group of aluminosilicates [42]. Interestingly, the incorporation of Mg into zeolite Y increased hydrophobicity Mg-Y-impr in comparison with parent Na-Y zeolite. This change corresponded with the porosity of the Mg-containing zeolite Y prepared via wet impregnation. The higher average pore size of the Mg-Y-impr sample (15.2 Å) compared to the parent sample Na-Y (11.5 Å) enabled easier water desorption from the zeolite surface due to lower diffusion limitation in pores.

Last but not least, we prepared reference samples without magnesium via the treatment of commercial zeolite Na-Y with pure distilled water under analogous conditions as in the case of impregnation, ion-exchange, and sonication. The interaction between the Na-Y sample and water under these conditions did not change the surface properties of such treated parent zeolites. The applied conditions turned out to be too mild to initiate any effect. Much more severe conditions for treating zeolite Y in aquatic environments were used by Latschka et al. [43], who treated zeolite Y with water at 200 °C and 42 bar in a flow reactor for 64 h. They indicated almost total crystallinity loss and significant microporosity destruction at high water flows. In turn, Ravenelle et al. [44] treated zeolite Y of various Si/Al ratios with liquid water at 150 and 200 °C under autogenic pressure. It was evidenced that treatment of zeolite Y with a Si/Al ratio higher than 14 resulted in the appearance of an amorphous phase. The observed amorphization increased with a rising Si/Al ratio and can be explained by the occurrence of hydrolysis of the siloxane bonds (Si-O-Si) dominating under steaming conditions.

## 4. Summary and Conclusions

In the present paper, we reported the impact of the choice of the specific method of the introduction of magnesium into the zeolite support (Mg-Y systems) mainly on their surface properties (morphology, roughness, crystallinity, and porosity). The applied techniques were impregnation, ion-exchange, and sonication. The results obtained from the AFM and CT analyses revealed a relatively smooth crystal surfaces across the parent zeolite sample without significant roughness. Similar observations were found for the modified zeolites (Mg-Y samples) prepared by both ion-exchange and sonication methods. Another effect was observed in the case of the Mg-Y sample obtained by a wet impregnation, which resulted in additional surface roughness. The observed effect might originate from the relatively long contact between zeolite and concentrated acidic magnesium nitrate solution (needed for the total evaporation of water from the impregnating medium). On the other hand, differentiated morphology and roughness may be correlated with the chemical form of magnesium present in Mg-Y samples. Significantly higher roughness of Mg-Y-impr than other studied samples corresponds to the oxide form of magnesium (MgO) existing in the external surface of this sample. Importantly, porosity studies conducted by low-temperature N_2_ sorption technique were in line with AFM, CT, and crystallinity analyses and confirmed the most apparent changes for Mg-Y prepared via the impregnation technique.

The obtained zeolite samples revealed high thermal stability, which could be helpful for their design as ingredients of energetic materials and their further application as functional materials in other industrial disciplines.

## Figures and Tables

**Table 1:** The surface roughness and crystallinity of the studied zeolite Y samples. Measurement errors were 5% and 15% for AMF and XRD calculations, respectively.

Sample	RMS [μm]	Crystallinity [Å]
Na-Y	0.189	897
Mg-Y-impr	0.235	677
Mg-Y-ion-exch	0.162	892
Mg-Y-son	0.156	833

**Table 2:** Effect on acid treatment conditions on dealumination efficiency of zeolite Y.

Sample	Agent	Concentration	Original Si/Al	Final Si/Al	Parent Zeolite	Reference
A_0.1_	HCl	0.1 mol/mL	5.0	6.7	H-Y	[30]
A_0.5_	HCl	0.5 mol/mL	5.0	8.6	H-Y	[30]
B_0.1_	EDTA	0.11 mol/mL	2.6	6.5	Na-Y	[31]

**Table 3:** Porosity data of the prepared samples. Measurement error was 5%.

Sample	S_Langmuir_, m^2^·g^−1^	d, [Å]	V_micro_, cm^3^·g^−1^	Total V_p_, cm^3^·g^−1^
Na-Y	1012	11.5	0.364	0.401
Mg-Y-impr	580	15.2	0.212	0.252
Mg-Y-ion-exch	866	13.4	0.313	0.346
Mg-Y-son	905	12.8	0.326	0.352

## Data Availability

The data presented in this study are available upon request from the corresponding authors Andrzej Biessikirski and Łukasz Kuterasiński.

## Appendix A

**Table A1:** Chemical composition of the studied samples. EDS measurement error was 1%. Partially adapted from [15] or calculated from [18] *.

Sample	Si/Al *	Si/Al [15]	Na [% wt.] [15]	Mg [%wt.] [15]
Na-Y	2.40	2.72	5.0	0.0
Mg-Y-impr	2.08	2.80	5.2	4.2
Mg-Y-ion-exch	2.22	2.23	1.8	2.7
Mg-Y-son	2.49	2.34	3.4	3.1
